# [Retracted] N-Myc downstream-regulated gene 2 suppresses the proliferation of T24 human bladder cancer cells via induction of oncosis

**DOI:** 10.3892/mmr.2025.13777

**Published:** 2025-12-15

**Authors:** Jie Huang, Zhou Wu, Guangxiu Wang, Yingxian Cai, Minshan Cai, Yaozhang Li

Mol Med Rep 12: 5730–5736, 2015; DOI: 10.3892/mmr.2015.4169

Following the publication of the above paper, a concerned reader drew to the Editor's attention that, within the left-hand and centre data panels of Fig. 6 on p. 5735, apparent anomalies were identifiable, including unexpectedly similar-looking cells and repeated patternings of these cells in terms of their layout/arrangement, albeit with inversions of the cells in certain cases. In addition, it was noted that some of the data featured in Table I and in Fig. 4B were strikingly similar to data that had previously appeared in a paper published in the journal *Cell Biochemistry and Biophysics* that was written by different authors at different research institutes.

After having conducted an independent investigation of this paper in the Editorial Office, the Editor of *Molecular Medicine Reports* has determined that it should be retracted from the Journal on account of a lack of confidence in the authenticity of the data. The authors were asked for an explanation to account for these concerns, but the Editorial Office did not receive a reply. The Editor regrets any inconvenience that has been caused to the readership of the Journal.

